# Sequencing and genotypic analysis of the triosephosphate isomerase (*TPI1*) locus in a large sample of long-lived Germans

**DOI:** 10.1186/1471-2156-9-38

**Published:** 2008-05-29

**Authors:** Markus Ralser, Almut Nebel, Rabea Kleindorp, Sylvia Krobitsch, Hans Lehrach, Stefan Schreiber, Richard Reinhardt, Bernd Timmermann

**Affiliations:** 1 Max Planck Institute for Molecular Genetics, Ihnestraße 73, 14195 Berlin, Germany; 2 Institute of Clinical Molecular Biology, Christian-Albrechts-University, Schittenhelmstraße 12, 24105 Kiel, Germany

## Abstract

**Background:**

Triosephosphate isomerase (TPI) is a central and conserved glycolytic enzyme. In humans, TPI is encoded by a single gene on 12p13, and associated with a rare genetic disorder, TPI deficiency. Reduced TPI activity can increase specific oxidant resistances of model organisms and TPI null-alleles have been hypothesized to promote a heterozygote advantage in man. However, comprehensive genetic information about the *TPI1 *locus is still lacking.

**Results:**

Here, we sequenced the *TPI1 *locus in a sample of 357 German long-lived individuals (LLI) aged 95 to 110 years. We identified 17 different polymorphisms, of which 15 were rare and previously unknown. The two remaining SNPs occurred at much higher frequency and were tested for association with the longevity phenotype in larger samples of LLI (n = 1422) and younger controls (n = 967). Neither of the two markers showed a statistically significant difference in allele or genotype frequency between LLI and control subjects.

**Conclusion:**

This study marks the *TPI1 *locus as extraordinarily conserved, even when analyzing intronic and non-coding regions of the gene. None of the identified sequence variations affected the amino acid composition of the TPI protein and hence, are unlikely to impact the catalytic activity of the enzyme. Thus, TPI variants occur less frequent than expected and inactive alleles are not enriched in German centenarians.

## Background

Triosephosphate isomerase (TPI, TIM; EC 5.3.1.1) is a cytoplasmic enzyme of the carbohydrate metabolism and catalyzes the glycolytic interconversion of the three-carbon sugars glyceraldehyde-3-phosphate and dihydroxyacetone phosphate in an extraordinarily efficient way [1,2]. In most species, this enzyme is present as a soluble, stable dimer and appears to be highly conserved among all kingdoms. In humans, the TPI enzyme is encoded by a single gene located at chromosome 12p13 [3]. This gene is associated with a rare genetic disorder, triosephosphate isomerase deficiency, initially described in 1965 [4], that is a unique glycolytic enzymopathy with autosomal recessive inheritance characterized by chronic haemolytic anaemia, cardiomyopathy, susceptibility to infections, severe neurological dysfunction, and, in most cases, death in early childhood [5,6]. The disease pathology correlates with a drastic decrease in the enzymatic activity of TPI and consequently, increased concentration of the TPI substrate dihydroxyacetone phosphate (reviewed in [6,7]). Analysis of the pathogenic mutations in a recombinant yeast model revealed that the decrease in TPI activity is not based on an *per se *inactive enzyme species, but likely the consequence of a misguided regulatory mechanism that might be caused by altered dimerization of the pathogenic TPI isoforms [8]. These results are in good agreement with previous observations made in mice showing that a complete lack of TPI activity (homozygous TPI null alleles) causes embryonic lethality at the earliest stages [9]. Accordingly, a recent study in *Drosophila *reveals that the pathogenesis of TPI deficiency could result from proteasomal degradation of the apparently functional enzyme [10].

Remarkably, in several human populations, unexpected high frequencies of individuals exhibiting about 50% of the normal TPI activity have been detected [11-15], indicating heterozygously inherited null alleles. The estimated allele frequencies of these ranged from 0.002 (Europeans) to 0.02 (Afro-Americans) [11-15], but the underlying molecular genetic defects have so far remained unknown. An association of reduced TPI activity with three allelic variants of the *TPI *promoter region (located at pos. -5, -8, and -24) in African-Americans [11] could not be confirmed in a follow-up study; these single nucleotide polymorphisms (SNPs) turned out to represent common haplotypes in another African-American sample [16]. Keeping in mind the aforementioned lethality of homozygous null alleles, the high frequency of the heterozygous null alleles may indicate an evolutionary advantage of a *TPI*^+/0 ^genotype [8,9,11]. The nature of this potential *heterozygote advantage *has not yet been elucidated, however, reduced TPI activity mediates cellular resistance to conditions of oxidative stress [8]. This phenotype has been studied in yeast and in *C. elegans*, and is based on a metabolic re-routing between glycolysis and the associated pentose phosphate pathway [17]. This observation is of basic biological interest since these two metabolic pathways are not only implicated in the cellular energy supply, but are also involved in cell growth, cell death and in the ageing process. Thus, the *TPI1 *locus represents an interesting target for genetic analysis, especially for studies focusing on ageing or oxidative-stress processes. However, at least for European populations, comprehensive sequence information about allelic *TPI *variants and their distributions and frequency is still lacking. Moreover, since oxidative stress is a key player in the ageing process – and model organisms with reduced TPI activity are more resistant to it – it is also of viable interest to ascertain the distribution and frequency of allelic *TPI *variants in humans who have attained an exceptional life span above average. Therefore, we re-sequenced the *TPI1 *locus in an extended sample of 357 German long-lived individuals aged 95 to 107 years. Two of the identified frequent polymorphisms were subsequently tested for association with the longevity phenotype.

## Methods

### Subjects

In this study, we sequenced the *TPI1 *locus from DNA samples of 357 German long-lived individuals (LLI) drawn from a larger collection [18]. The study participants had a mean age of 97.2 years at ascertainment ranging from 95 to 107 years; about 71% of them were female.

The subsequent association analyses were performed on the entire collection comprising 1422 LLI (mean age: 98.8 years, age range: 95–110 years) and 967 younger controls (mean age: 66.8 years, ranging from 60 to 75 years). The cases were specifically matched to the controls by ethnicity, gender and geographic origin within Germany. The DNA collections and the recruitment procedures were reported in detail elsewhere [18]. DNA was isolated from blood samples of all participants using standard methods. All subjects gave written informed consent prior to participation. The study was approved by the Ethics Committee of the University Hospital Schleswig-Holstein in Kiel.

### Analysis of genetic variation by re-sequencing

The *TPI1 *locus including all introns and exons was dissected into suitable polymerase-chain-reaction (PCR) fragments that ranged in size from 454 to 800 bps (details of PCR are provided in Supplementary Material). All primers were designed with Primer3 and all resulting fragments were amplified by PCR with Taq DNA Polymerase (total reaction volume, 20 μl) supplemented with a home-made PCR enhancer as described [19]. Both strands were routinely amplified and sequenced to ensure maximal accuracy in variation analysis. PCR primers were also used as sequencing primers. Additional internal primers were used for PCR products longer than 600 bp to ensure that there was double-stranded sequence information for the whole PCR fragment. Sequencing was performed on ABI3730xl automated DNA sequencers with the BigDye Terminator V3.1 Cycle Sequencing Kit (Applied Biosystems). The complete coding, flanking 5' and 3' untranslated regions of *TPI1 *and 100 bp of the promoter region were sequenced and compared with the reference sequence (GenBank accession number U47924).

Polyphred software together with Phred, Phrap, RepeatMasker, and Consed were used to detect polymorphisms. Of the 357 DNA samples, all genotypes (92.4 to 96.6%) at each polymorphic site of *TPI1 *were determined with maximal accuracy and included in the analysis.

### Genotyping

The SNP rs2071069 was genotyped using the SNPlex™ Genotyping System and SNP rs2071065 (hCV15868213) with a TaqMan^® ^SNP Genotyping Assay (Applied Biosystems, Foster City, USA). Single-marker case-control association analyses were performed with the software program Genomizer [20].

## Results and Discussion

**Figure 1:**
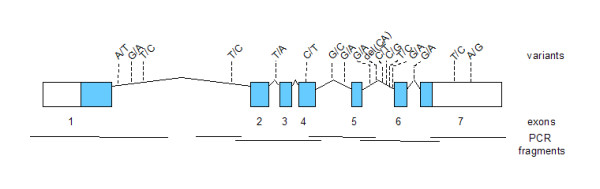
Genomic organization and polymorphic spectrum of *TPI1 *gene.

This study presents the first molecular analysis of the genetic variability of the human *TPI1 *gene locus in a large set of long-lived individuals. As illustrated in figure 1, the *TPI1 *locus is composed of 7 exons and spans a genomic region of 3.2 kb. This allowed high sequence coverage by using only 6 PCR fragments (please see Additional file 1 for the primer sequences used). We detected 17 different mutations or polymorphisms in the analyzed region of the *TPI1 *gene relative to the reference sequence (Table 1). Only two of them (rs2071065 and rs2071069) were previously known, whereas the other 15 were not identified in a dbSNP search. Out of these, 15 SNPs were rare, in fact, 11 variants occurred only once. Of note, one subject had three of these private mutations. None of the previously detected SNPs in African-Americans [11] were seen in the German sample.

**Table 1:** Mutations in the *TPI1 *gene identified among 357 long-lived individuals. Positions correspond to reference sequence (GenBank accession number U47924)

**Ref. pos.**	**Variation**	**Flanking site**	**Location**	**Allele Freq.**	**Status**
79766	A/T	CAGCGCCCTCTCCCG A GGCCCCGAGGCCCCG	intron 1	0.004	unknown
79834	G/A	GACGAGGGCCGCTGG G GTCCGGGCAGGGGCC	intron 1	0.002	unknown
79915	T/C	ATGCCCCTTGGACTA T GGGGCAGGTAAGGAC	intron 1	0.270	rs2071065
80568	T/C	TTGCTCCCTGGAGAA T GCTGAGTCTGTGAGG	intron 1	0.001	unknown
81046	T/A	CTTCCCTCACTTTCC T CGTTGAGGGGAAAGC	intron 2	0.001	unknown
81309	C/T	TCTGGCAGAGGGACT C GGAGTAATCGCCTGC	exon 4	0.003	unknown
81549	G/C	TTGCTTGGGGCCTAT G ACTTCTCCAGCCCCA	intron 4	0.001	unknown
81643	G/A	ACTCCGGAGAACCTG G CTGGAGAGCTCTTTC	intron 4	0.001	unknown
81856	G/A	ACACAGCCCACATGG G GCAACCCCTTATTTC	intron 5	0.260	rs2071069
81879	del(CA)	CTTATTTCAAAGACA CA GAGACCTTGAACCCA	intron 5	0.001	unknown
81959	C/T	CAGAGCCCTGGTACT C TGACTCAGTCAGAAA	intron 5	0.001	unknown
81980	C/G	CAGTCAGAAACCACA C TAAGTGTCCACTGGT	intron 5	0.025	unknown
81985	T/C	AGAAACCACACTAAG T GTCCACTGGTGCCAG	intron 5	0.001	unknown
82045	G/A	GTCTTACTTAGGCCA G CTTCTTGTTCTAGGC	intron 5	0.001	unknown
82214	G/A	GCCCTCGGACATGGA G GTGGGGATGGGGCAG	intron 6	0.001	unknown
82813	T/C	TATGTGAACCACCCA T GTGAGGGAATAAACC	exon 7	0.001	unknown
82860	A/G	GGTTTGTCTGCCTTC A CTGGACTTGCCCAGA	exon 7	0.001	unknown

As listed in Table 1, the two known *TPI1 *variants (rs2071065 and rs2071069) had a much higher allele frequency (0.270 and 0.260, respectively) compared to the others. One of these (SNP rs2071069) has been studied previously (termed TPI 2898 in [21] and TPI 2262 in [22]). Although its low frequency in the random sample, this SNP was found in all subjects having inherited the most common disease allele (TPI 1591C, encoding TPI_Glu104Asp_). This discovery allowed the authors to conclude that all TPI_Glu104Asp _subjects are descendants of a common ancestor that lived in today's France or England more than 1000 years ago [21,22].

The detected allele-frequencies of rs2071065 and rs2071069 prompted us to test whether there is a genetic association between the SNP alleles and the longevity phenotype in larger samples of LLI (n = 1422) and control individuals (n = 967). The genotype analyses revealed SNP frequencies that were quite similar to those determined by sequencing. SNP rs2071065 showed an allele frequency of 0.30 in the LLI and 0.28 in the controls, respectively; the second SNP rs2071069 had a frequency of 0.29 in the LLI and 0.27 in controls. However, neither of the two markers showed a statistically significant difference in allele or genotype frequency between LLI and control subjects, using the entire collection or subsamples stratified for gender (P > 0.05).

Fourteen of the detected mutations were located in intronic and three in exonic regions. However, none of the detected variants seem to affect the amino acid sequence of the TPI enzyme. The only SNP located within the *TPI1 *coding sequence (ref. pos. 81309) encodes Leu121 and is silent; and hence, none of the identified sequence variants is likely to result in a *TPI1 *null allele. This result seems to be inconsistent with a previous study based on a German population, in which 3000 persons were screened for reduced TPI activity; 3.7 per 1000 individuals showed reduced triosephosphate isomerase activity (in the range of 39 to 76% compared to the enzyme activity of controls) and were verified to be heterozygous carriers [14]; even higher frequencies of haploid TPI deficiencies were detected in other populations [11-15]). However, there are several possible explanations for the discrepancy of these biochemical screenings with the results of our sequence analyses. First, it is feasible that the number of sequenced individuals was too small to detect the predicted null alleles. Second, the reduced enzyme activities measured in the previous studies could be, at least in part, based on factors acting is *trans*; e.g. mutations affecting the activity of transcription factors controlling the expression of the TPI enzyme. Indeed, in the Eber et al. study, none of the heterozygous persons expressed an electrophoretic variant of the TPI enzyme [14]. Finally, it cannot be excluded from our data that inactive TPI alleles have negative effects on human life expectancy and hence, would be depleted in the analyzed LLI group. In fact, a variety of genes increasing the oxidative-stress resistances in model organisms can, dependent on environmental conditions, shorten their lifespan, and yeast as well as *C. elegans *with reduced TPI activity show indeed shortened lifespan when growing under standard laboratory conditions [17]. This phenotype might be caused by an observed shift of the cellular redox potential versus its reduced state [17], resulting in a condition termed reductive stress. Remarkably, reductive stress has been found to be deleterious and disease-causing in mice [23]. Nonetheless, our data reveals no enrichment of *TPI *null alleles among long-lived individuals, indicating that the potential *heterozygote advantage *of the *TPI*^+/0 ^genotype does not promote longevity in humans.

For a final answer to all these questions, further studies have to focus on much larger numbers of studied individuals and on the comparison of *TPI *allele frequencies in different age groups and populations. Fortunately, next generation sequencing technology (e.g. massively parallel pyrosequencing with 454/Roche FLX system, sequencing by synthesis with the Illumina/Solexa system or sequencing by ligation with the Applied Biosystems SOLiD system) will allow coping with the required sample quantities at affordable costs.

## Conclusion

In this study, we analyzed and sequenced the *TPI1 *locus in a large sample of LLI of German descent. We identified 17 SNPs of which 15 were rare and previously unknown. None of the identified sequence variations affected the amino acid composition of the TPI protein and hence, these mutations are unlikely to impact the catalytic activity of the enzyme. Thus, genetic TPI variants occur less frequent than expected and inactive TPI alleles are not enriched in German centenarians.

## Competing interests

The authors declare that they have no competing interests.

## Authors' contributions

BT and RR conducted and designed the re-sequencing, AN and RK the genotyping, BT and AN the statistical analyses, MR, SK, HL and BT conceived and designed the study, SS conceived and organized the sampling, MR wrote the first manuscript draft, MR, SK, AN and BT the final version. All authors read and approved the final manuscript.

## Supplementary Material

Additional file 1 Supplementary Table. PCR primer sequences. Click here for file

## References

[B1] Trentham DR, McMurray CH, Pogson CI (1969). The active chemical state of D-glyceraldehyde 3-phosphate in its reactions with D-glyceraldehyde 3-phosphate dehydrogenase, aldolase and triose phosphate isomerase. Biochem J.

[B2] Putman SJ, Coulson AF, Farley IR, Riddleston B, Knowles JR (1972). Specificity and kinetics of triose phosphate isomerase from chicken muscle. Biochem J.

[B3] Brown JR, Daar IO, Krug JR, Maquat LE (1985). Characterization of the functional gene and several processed pseudogenes in the human triosephosphate isomerase gene family. Mol Cell Biol.

[B4] Schneider AS, Valentine WN, Hattori M, Heins HL (1965). Hereditary Hemolytic Anemia with Triosephosphate Isomerase Deficiency. N Engl J Med.

[B5] Schneider AS (2000). Triosephosphate isomerase deficiency: historical perspectives and molecular aspects. Baillieres Best Pract Res Clin Haematol.

[B6] Orosz F, Olah J, Ovadi J (2006). Triosephosphate isomerase deficiency: facts and doubts. IUBMB Life.

[B7] Olah J, Orosz F, Keseru GM, Kovari Z, Kovacs J, Hollan S, Ovadi J (2002). Triosephosphate isomerase deficiency: a neurodegenerative misfolding disease. Biochem Soc Trans.

[B8] Ralser M, Heeren G, Breitenbach M, Lehrach H, Krobitsch S (2006). Triose phosphate isomerase deficiency is caused by altered dimerization--not catalytic inactivity--of the mutant enzymes. PLoS ONE.

[B9] Merkle S, Pretsch W (1989). Characterization of triosephosphate isomerase mutants with reduced enzyme activity in Mus musculus. Genetics.

[B10] Seigle JL, Celotto AM, Palladino M (2008). Degradation of functional TPI protein underlies sugarkill pathology. Genetics.

[B11] Watanabe M, Zingg BC, Mohrenweiser HW (1996). Molecular analysis of a series of alleles in humans with reduced activity at the triosephosphate isomerase locus. Am J Hum Genet.

[B12] Mohrenweiser HW, Wurzinger KH, Neel JV (1987). Frequency and distribution of rare electrophoretic mobility variants in a population of human newborns in Ann Arbor, Michigan. Ann Hum Genet.

[B13] Mohrenweiser HW, Fielek S (1982). Elevated frequency of carriers for triosephosphate isomerase deficiency in newborn infants. Pediatr Res.

[B14] Eber SW, Dunnwald M, Heinemann G, Hofstatter T, Weinmann HM, Belohradsky BH (1984). Prevalence of partial deficiency of red cell triosephosphate isomerase in Germany--a study of 3000 people. Hum Genet.

[B15] Neel JV, Satoh C, Goriki K, Asakawa J, Fujita M, Takahashi N, Kageoka T, Hazama R (1988). Search for mutations altering protein charge and/or function in children of atomic bomb survivors: final report. Am J Hum Genet.

[B16] Schneider A, Forman L, Westwood B, Yim C, Lin J, Singh S, Beutler E (1998). The relationship of the -5, -8, and -24 variant alleles in African Americans to triosephosphate isomerase (TPI) enzyme activity and to TPI deficiency. Blood.

[B17] Ralser M, Wamelink MM, Kowald A, Gerisch B, Heeren G, Struys EA, Klipp E, Jakobs C, Breitenbach M, Lehrach H, Krobitsch S (2007). Dynamic rerouting of the carbohydrate flux is key to counteracting oxidative stress. J Biol.

[B18] Nebel A, Croucher PJ, Stiegeler R, Nikolaus S, Krawczak M, Schreiber S (2005). No association between microsomal triglyceride transfer protein (MTP) haplotype and longevity in humans. Proc Natl Acad Sci U S A.

[B19] Ralser M, Querfurth R, Warnatz HJ, Lehrach H, Yaspo ML, Krobitsch S (2006). An efficient and economic enhancer mix for PCR. Biochem Biophys Res Commun.

[B20] Franke A, Wollstein A, Teuber M, Wittig M, Lu T, Hoffmann K, Nurnberg P, Krawczak M, Schreiber S, Hampe J (2006). GENOMIZER: an integrated analysis system for genome-wide association data. Hum Mutat.

[B21] Arya R, Lalloz MR, Bellingham AJ, Layton DM (1997). Evidence for founder effect of the Glu104Asp substitution and identification of new mutations in triosephosphate isomerase deficiency. Hum Mutat.

[B22] Schneider A, Westwood B, Yim C, Cohen-Solal M, Rosa R, Labotka R, Eber S, Wolf R, Lammi A, Beutler E (1996). The 1591C mutation in triosephosphate isomerase (TPI) deficiency. Tightly linked polymorphisms and a common haplotype in all known families. Blood Cells Mol Dis.

[B23] Rajasekaran NS, Connell P, Christians ES, Yan LJ, Taylor RP, Orosz A, Zhang XQ, Stevenson TJ, Peshock RM, Leopold JA, Barry WH, Loscalzo J, Odelberg SJ, Benjamin IJ (2007). Human alpha B-crystallin mutation causes oxido-reductive stress and protein aggregation cardiomyopathy in mice. Cell.

