# Supplementary material for: Sequencing and genotypic analysis of the triosephosphate isomerase (TPI1) locus in a large sample of long-lived Germans
Source: BMC Genet. 2008 May 29;9:38. doi: 10.1186/1471-2156-9-38 (PMC2424074; doi:10.1186/1471-2156-9-38)
Supplement: Additional file 1 — Supplementary Table. PCR primer sequences. [file 1471-2156-9-38-S1.doc]

**Supplementary Material**

**Supplementary Table 2. PCR primer sequences**

| Name | Sequence (5’-3’) | Fragments |
| --- | --- | --- |
| TPI1-1F | GCCTGCTCAGCAGTCGGGCATG | 1 |
| TPI1-2R | AAGGCGGGAGTCGTTCAGGTT |
| TPI1-3F | GCCGCACGTAGCCCCAGAC | 2 |
| TPI1-4R | TTCCCCACAATTCCTAACCAG |
| TPI1-5F | GCCGTGGCCTCTCAGGGGTATC | 3 |
| TPI1-6R | CTGAGTCTCTGTGGCCCTG |
| TPI1-7F | ATTGGGGAGAAGCTAGATGAAAG | 4 |
| TPI1-8R | CAAGAAGCTGGCCTAAGTAAGACC |
| TPI1-9F | CCTCATCCCAGCCTGCCTC | 5 |
| TPI1-10R | TTACTGGGCTTCTGGGCTGC |
| TPI1-11F | CAAGCCCGAATTCGTGGAC | 6 |
| TPI1-12R | ACCCAAGCTCTGCGGAAATC |
